# Delirium due to Trousseau syndrome treated with memantine and perospirone: A case report

**DOI:** 10.1002/pcn5.159

**Published:** 2023-11-16

**Authors:** Junji Yamaguchi, Takatoshi Hirayama, Ryoichi Sadahiro, Rika Nakahara, Hiromichi Matsuoka

**Affiliations:** ^1^ Asakusa Family Clinic Tokyo Japan; ^2^ Department of Psycho‐Oncology National Cancer Center Hospital Tokyo Japan

**Keywords:** cancer, delirium, memantine, perospirone, Trousseau syndrome

## Abstract

**Background:**

Trousseau syndrome is a hypercoagulability syndrome associated with cancer. It is known that delirium occasionally occurs after the onset of Trousseau syndrome. However, there have been no detailed reports about treatment for psychiatric symptoms of delirium associated with Trousseau syndrome.

**Case Presentation:**

A 61‐year‐old man with lung cancer was hospitalized due to Trousseau syndrome. Delirium occurred after hospitalization and psychiatric symptoms worsened. Although haloperidol, risperidone, and chlorpromazine were used, severe insomnia persisted. After memantine (5 mg/day) was used with perospirone, the patient's psychiatric symptoms gradually decreased; he could sleep for 4–5 h at night. Due to psychiatric improvement, he was able to return home and resume immunotherapy for lung cancer as scheduled.

**Conclusion:**

We report the first case of Trousseau syndrome delirium treated by memantine used with perospirone. Although further studies are needed, memantine and perospirone might be candidates for the management of psychiatric symptoms associated with Trousseau syndrome.

## Graphical abstract

We report the first case of Trousseau syndrome delirium treated by memantine used with perospirone. Memantine and perospirone might be candidates for the management of psychiatric symptoms associated with Trousseau syndrome

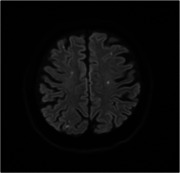

## BACKGROUND

Trousseau syndrome is a clinical condition named after Armand Trousseau, who first reported the relationship between venous thromboembolism and malignancy in 1865.^1^ Today, Trousseau syndrome is defined as unexplained thrombotic events that precede the diagnosis of an occult visceral malignancy or appear concomitantly with the tumor.^2^ The clinical manifestations of Trousseau syndrome in patients with cancer include various conditions, such as deep vein thrombosis, pulmonary embolism, and brain infarction.^3^ However, there have been almost no reports about delirium due to Trousseau syndrome. Moreover, to the best of our knowledge based on the literature review, there are no previous reports about psychiatric symptoms or management of delirium associated with Trousseau syndrome. Furthermore, we could find no reports mentioning how often delirium occurs with Trousseau syndrome. Although antipsychotics could be used off‐label for delirium in clinical situations, we did not find any reports on the effectiveness of these drugs. Here, we describe a first case of Trousseau syndrome in which the psychiatric symptoms of delirium were controlled by memantine, used with perospirone.

## CASE PRESENTATION

**Figure 1:**
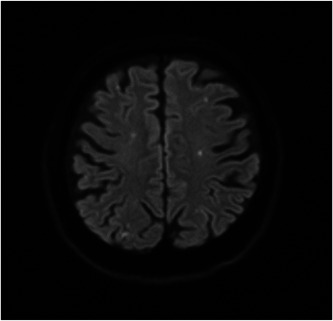
Magnetic resonance image of the head taken at on hospitalization day. There are multiple small acute brain infarctions distributed in both hemispheres indicating Trousseau syndrome.

A 61‐year‐old man with stage IVB pulmonary adenocarcinoma for more than 2 years was admitted to our hospital due to sudden onset of impaired consciousness, left hemiplegia, left facial nerve paralysis, and dysarthria. Vital signs were as follows: temperature 36.9°C, pulse 74 beats/min, blood pressure 160/104 mmHg, and SpO_2_ 98%. He had a history of diabetes but no history of hypertension or atrial fibrillation. Diffusion‐weighted magnetic resonance imaging revealed many small acute brain infarctions in both hemispheres (Figure 1). The patient's symptoms were consistent with Trousseau syndrome secondary to lung cancer. His mental status had been otherwise normal. He had no history of mental disorders and he had never had cognitive problems. With the introduction of anticoagulation therapy (days 0–10, heparin 23,000 U/day; day 11 onwards, edoxaban tosylate hydrate 60 mg/day), his neurological symptoms recovered. Several days after hospital admission, neurological deficits had resolved, except for mild left arm paralysis and left facial nerve paralysis.

However, delirium occurred after Trousseau syndrome 1 day after hospital admission. Blood test was unremarkable. His daily medications included loxoprofen (180 mg/day, oral), lansoprazole (15 mg/day, oral), and suvorexant (15 mg/day, oral), which were less likely to induce delirium. Moreover, the last pembrolizumab (anti‐programmed cell death 1 antibody [anti PD‐1]) was given 4 months ago and the possibility of psychiatric disorders including delirium associated with anti PD‐1 is reported to be 1.91% and is the lowest in immune checkpoint inhibitors.^4^ We therefore diagnosed that this case was delirium due to Trousseau syndrome and delirium due to other causes was deniable.

**Figure 2:**
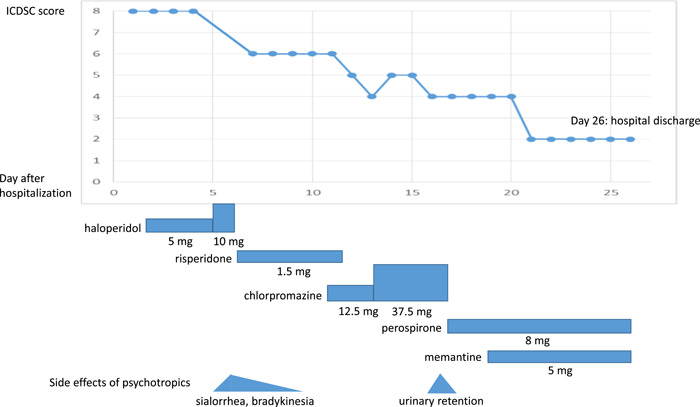
The clinical course of delirium with Trousseau syndrome and psychotropics. ICDSC, Intensive Care Delirium Screening Checklist.

Psychiatric symptoms such as insomnia, agitation, and restlessness gradually progressed to the extent that the patient moved around his room restlessly at night without sleeping. He could not stay still, restlessly sitting and standing beside his bed. He often got confused and admitted the existence of visual hallucination. We first selected an intravenous drip of haloperidol or risperidone liquid because the patient admitted difficulty in swallowing after Trousseau syndrome, and effects such as sedation and antihallucination were expected. Haloperidol (days 2–6, up to 10 mg/day, intravenous), risperidone liquid (days 6–12, up to 1.5 mg/day, oral), and chlorpromazine (days 11–17, up to 37.5 mg/day, oral) were used to manage his psychiatric symptoms (Figure 2). Before administration, it was explained to the patient and his family that these were off‐label prescriptions and there were possible side effects. After the initiation, hallucination and agitation were gradually lessened. However, inattention, altered sleep/wake cycle, altered level of consciousness, and symptom fluctuation persisted. The patient complained of unbearable insomnia for more than 2 weeks. In addition, extrapyramidal side effects such as sialorrhea and bradykinesia were observed. Moreover, chlorpromazine was intolerable because of drowsiness and urinary retention.

We then tried perospirone (days 17 onwards, 8 mg/day, oral) but this was not effective either and insomnia persisted. The Intensive Care Delirium Screening Checklist (ICDSC)^5^ score at that time remained 4, indicating the existence of delirium, and hospital discharge remained impossible. We maintained the use of perospirone at this point because we began to consider the combined use of perospirone and another medication other than antipsychotics.

**Figure 3:**
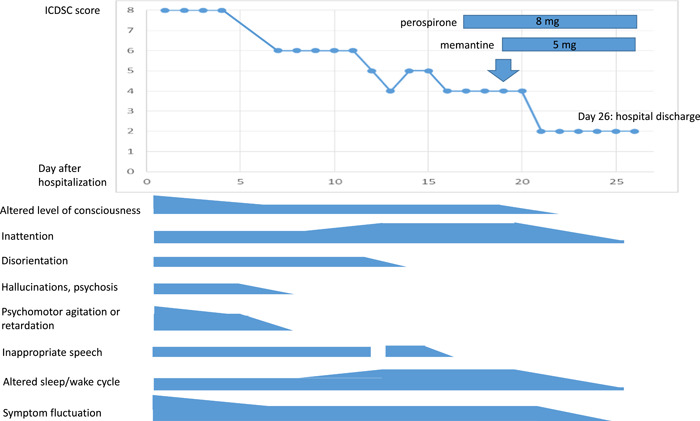
The psychiatric change of delirium with Trousseau syndrome. ICDSC, Intensive Care Delirium Screening Checklist.

After the failure of the antipsychotics, we tried memantine to alleviate his psychiatric symptoms. We considered that was worth trying because memantine has different mechanisms of action from antipsychotics and it has no drug interactions with anticancer agents,^6^ which is favorable in terms of not hindering cancer treatment. Before use, we fully explained to the patient and his family that this was an off‐label prescription and also explained the clinical effects and possible side effects. After the use of memantine (days 19 onwards, 5 mg/day, oral) with perospirone, the patient's psychiatric symptoms lessened. He could sleep for 4–5 h at night for the first time after admission. He could rest in his room at night and restlessness gradually resolved. Moreover, daytime sleepiness was not observed. Memantine was tolerated with no apparent side effects. With psychiatric improvement, he was able to return home after 26 days of hospitalization (Figure 3). Pembrolizumab was restarted regularly as previously planned, with the aid of his family and his home‐visiting physician.

## DISCUSSION AND CONCLUSION

As far as we know, there have been no reports about treatment and drug choice for the psychiatric symptoms of delirium due to Trousseau syndrome. This is the first case report of Trousseau syndrome delirium alleviated by memantine used with perospirone. It was notable that antipsychotics used as the sole regimen were effective for hallucinations and psychomotor agitation, but not especially effective for inattention and persisting insomnia in Trousseau syndrome delirium.

Perospirone is an atypical antipsychotic with potent serotonin 5‐hydroxytryptamine 2  and dopamine D2 antagonist activity and Takeuchi et al. showed the effectiveness of perospirone for delirium treatment.^7^ They reported that perospirone was effective in 86.8% (33/38) of patients, and the effect appeared within several days (5.1 ± 4.9 days). The initial dose was 6.5 ± 3.7 mg/day and the maximum dose of perospirone was 10.0 ± 5.3 mg/day. However, the effect of perospirone for Trousseau syndrome delirium was not mentioned, in contrast to delirium due to other causes.

In this case, we used perospirone at first and then used memantine simultaneously. It is possible that the sole use of perospirone contributed to the alleviation of Trousseau syndrome delirium. However, when perospirone was used without memantine, there was no apparent psychiatric improvement. In addition, perospirone 8 mg is regarded as equivalent to haloperidol 2 mg and risperidone 1 mg.^8^ We used haloperidol up to 10 mg and risperidone up to 1.5 mg, both of which were ineffective to inattention, altered sleep/wake cycle, and altered level of consciousness. We therefore consider that not only perospirone but also memantine contributed to the alleviation of psychiatric symptoms of Trousseau syndrome delirium.

We speculate that the reason why memantine alleviated the delirium due to Trousseau syndrome might be partly related to the recovery of N‐methyl‐D‐aspartate (NMDA) receptors, which are hypothesized to play a role in psychiatric symptoms such as the positive and negative symptoms of schizophrenia and cognitive function.^9^ Memantine is thought to work as a partial antagonist and a partial agonist of the NMDA receptor.^10^ Thus, memantine might contribute to the recovery of NMDA receptor function, thereby reducing the psychiatric symptoms of delirium due to Trousseau syndrome, as in schizophrenia. In addition, memantine is reported to be an effective augmentation agent in refractory schizophrenia using clozapine,^11^, ^12^ therefore it is possible that the combination of memantine with antipsychotics, including perospirone, may also be effective for psychiatric symptoms of delirium following Trousseau syndrome.

We also speculate that the mechanism by which memantine alleviates the psychiatric symptoms of delirium is related to the attenuation of progression of ischemic changes and brain infarction at the microscopic level. Chen et al. showed that memantine could prevent ischemic stroke‐induced neurological deficits and brain infarction both in vivo and in vitro, which attenuates brain damage and neuronal loss in rats.^13^ Memantine is expected to induce the same results in Trousseau syndrome, in which ischemic changes due to the progression of microcoagulation by tumor cells are predominant. Besides, as for the rapid improvement, memantine is reported to have effects on brain infarction within 5 days by lowering matrix metalloproteinases‐9, which is associated with the development of delirium.^14^, ^15^ We speculate that the stroke‐related mechanism by memantine more strongly contributed in this case because it is not dominant in chronic dementia, but is expected to occur abundantly in Trousseau syndrome.

Based on the hypothesis that memantine is effective for delirium with Trousseau syndrome, is memantine also effective for delirium due to other causes? There are a few reports of good and a few reports of bad effects of memantine in delirium.^16^, ^17^, ^18^ Although the neuropathogenesis of delirium remains unclear, acetylcholine deficiency and dopamine and glutamate excess are related to the onset of delirium.^19^ As for glutamate, Choi mentioned that glutamate neurotoxicity has been linked to a lethal influx of extracellular Ca^2+^ through cell membrane channels,^20^ pointing out that an influx of extracellular Ca^2+^ can cause cell death and Ca^2+^ is associated with excessive NMDA receptor activation. Based on the NMDA receptor hypothesis, it is possible that memantine is also effective for delirium due to other causes. To answer this question, we think that it is necessary to investigate the effectiveness of memantine for delirium due to other causes as well as delirium due to Trousseau syndrome and compare their differences.

This case study has several limitations. First, we only examined one case. Second, it could be that this case was a part of transit syndrome induced by Trousseau syndrome. However, psychiatric symptoms such as sleep cycle and inattention were unchanged or worse before the introduction of memantine and perospirone. The ICDSC score fell below 4 after the start of memantine and we suggest that natural course alone may not be enough to explain the whole psychiatric improvements. Third, other factors such as nursing care and environmental changes after hospitalization might have influenced the patient's mental status, therefore, to validate the effect of memantine and perospirone, it is necessary to increase the number of clinical cases where memantine and perospirone are used to treat delirium due to Trousseau syndrome while regulating other factors such as psychotropics, environment, and nursing care.

Memantine and perospirone are regarded as possible candidates for the management of psychiatric symptoms associated with Trousseau syndrome. Further investigations are necessary to verify their clinical effect.

## AUTHOR CONTRIBUTIONS

Junji Yamaguchi and Takatoshi Hirayama treated the patient and acquired data. Junji Yamaguchi drafted the manuscript. Takatoshi Hirayama supervised the work. All authors substantially revised the manuscript. All authors read and approved the final manuscript.

## CONFLICT OF INTEREST STATEMENT

The authors declare no conflict of interest.

## ETHICS APPROVAL STATEMENT

The ethics committee is not required to review case reports. We conducted this study in accordance with the Helsinki Declaration and its later amendments or comparable ethical standards.

## PATIENT CONSENT STATEMENT

The patient has provided written consented for the submission of this case report to the journal.

## CLINICAL TRIAL REGISTRATION

N/A.

## Data Availability

The data are not available due to privacy restrictions.
